# Thermally enhanced solid–liquid separation process in food waste biorefinery: modelling the anaerobic digestion of solid residues

**DOI:** 10.3389/fbioe.2024.1343396

**Published:** 2024-02-02

**Authors:** Agata Gallipoli, Francesca Angelini, Stefania Angelini, Camilla Maria Braguglia, Daniele Montecchio, Barbara Tonanzi, Andrea Gianico

**Affiliations:** National Research Council of Italy, Water Research Institute, CNR-IRSA, Rome, Italy

**Keywords:** food waste, biorefinery, side-stream valorization, resource recovery, biogas, 16S RNA sequencing, ADM1

## Abstract

The biochemical valorization potential of food waste (FW) could be exploited by extracting decreasing added-value bio-based products and converting the final residues into energy. In this context, multi-purpose and versatile schemes integrating thermal and biochemical conversion processes will play a key role. An upstream thermal pretreatment + solid-liquid separation unit was here proposed to optimize the conversion of the liquid fraction of FW into valuable chemicals through semi-continuous fermentation process, and the conversion of the residual solid fraction into biomethane through anaerobic digestion. The solid residues obtained after thermal pretreatment presented a higher soluble COD fraction, which resulted in higher methane production with respect to the raw residues (0.33 vs. 0.29 Nm^3^CH_4_ kg^-1^VS_fed_) and higher risk of acidification and failure of methanogenesis when operating at lower HRT (20d). On the contrary, at HRT = 40 d, the pretreatment did not affect the methane conversion rates and both tests evidenced similar methane productions of 0.33 Nm^3^CH_4_ kg^-1^VS_fed_. In the reactor fed with pretreated residue, the association of hydrogenotrophic methanogens with syntrophic bacteria prevented the acidification of the system. Modelling proved the eligibility of the FW solid residues as substrates for anaerobic digestion, given their small inert fractions that ranged between 0% and 30% of the total COD content.

## 1 Introduction

By 2050, worldwide municipal solid waste (MSW) generation is expected to rise up to 3.4 billion metric tons, and this is increasingly becoming a global major issue. However, less than 20% of waste is recycled annually, with huge quantities still sent to landfills (Kaza et al., 2018).

The biodegradable fraction, called Organic Fraction of Municipal Solid Waste (OFMSW), may account for up to 50% of the total MSW and it represents a major cause of greenhouse gases emissions, soil and water pollution, and loss of nutrients in the environment (Mak et al., 2020).

The OFMSW is mainly comprised of household and restaurant food waste (FW). The composition of FW typically varies depending on geographical and seasonal conditions, thus making FW treatment and disposal a complex issue to deal with (Arvanitoyannis, 2008; Braguglia et al., 2018; Marin et al., 2022). On the other hand, FW valorization play an essential role against exhaustion of non-renewable natural resources and is instrumental for reducing the environmental and economic burden of FW and transitioning to a circular economy (Coma et al., 2017; Braguglia et al., 2018). Usually, FW is high in moisture content, which provides beneficial effects in some of the conversion processes such as anaerobic digestion (AD), fermentation, hydrolysis, hydrothermal carbonization. However, sometimes size reduction or pretreatment is necessary to improve the conversion process and extract precursors to value-added products (Roy et al., 2023).

EU has adopted targets to achieve a 32% share of renewable energy in energy consumption by 2030, and methane from organic waste will play an important role. Refining waste to biomethane has been widely adopted in EU, especially in Germany, France and Sweden, in which the numbers of biomethane injection plants raised in the last decade (Stürmer, 2020). Besides EU, utilization of FW streams in waste-to-energy technologies has increased worldwide over the years.

Anaerobic bioprocesses are considered good alternatives for the management of organic waste, due to their prospects for resource and energy recovery with limited environmental footprints (Capson-Tojo et al., 2016; Roy et al., 2023). Several factors have been investigated for the optimization of biogas recovery, including operative conditions of AD (Temperature, C:N ratio, pH, organic loading rate and hydraulic retention time), pretreatments, reactor design, and co-digestion with other feedstocks (Braguglia et al., 2018; Hunter et al., 2021).

Various studies have also investigated the dynamics and the role of the biomass in the AD process conducted used FW as feed (Zamanzadeh et al., 2016; Zhang et al., 2020; Li et al., 2022; Sharma et al., 2023). As well known, the AD microbiome included a mix of fermentative, hydrolytic and acidogenic bacteria, but also hydrogentrophic and acetoclastic methanogens (De Groof et al., 2020). Indeed, AD systems are characterized by a high level of microbial diversity generally investigated by high-throughput sequencing analysis of 16S RNA. Bacteroidetes, Chloroflexi, Firmicutes and Proteobacteria are the main bacterial phyla generally present as abundant groups in reactors (Guo et al., 2014; Zamanzadeh et al., 2016; De Jonge et al., 2017; Tonanzi et al., 2018; Tonanzi et al., 2020; Tonanzi et al., 2021).

Methanogenic pathway is often hindered in FW AD process. As previously reported, methanogens are known to be slow growing and sensitive to environmental conditions, such as high concentrations of volatile fatty acids and ammonia, and their community structure is critical to avoid system acidification (Capson- Tojo et al., 2018). Due to their high substrate affinity, acetoclastic methanogens such as Methanosaeta are generally predominant under unstressed conditions and consequently acetotrophic methanogenesis is the predominant pathway for methane production in these systems. However, under stressful conditions of AD, these methanogens are preferentially inhibited, and hydrogenotrophic and mixotrophic microorganisms, capable of consuming hydrogen or both acetate and hydrogen to produce methane, such as Methanobacterium, Methanococcus, Methanobrevibacter, Methanomicrobium, and Methanosarcina, become predominant (De Vrieze et al., 2012; Venkiteshwaran et al., 2016; Capson-Tojo et al., 2018). Complexity arises, however, due to the number of possible metabolic routes the AD process can take, and this can impact the biogas yield due to the formation of other intermediates (Hunter et al., 2021).

Mathematical modelling has been widely adopted in the literature to achieve a better comprehension of the complexity of the anaerobic processes. In particular, the Anaerobic Digestion Model n°1 (ADM1), developed by the International Water Association (Batstone et al., 2002), is being regarded worldwide as the standard model for both researchers and practitioners. Modelling has been applied to a large diversity of substrates; in fact, although originally intended for sludge, the ADM1 has proved to be suitable for basically all the anaerobically biodegradable substrates (Batstone et al., 2015), including FW. Several applications have been presented in the literature, as, for example, modelling aimed at assessing the effects of thermal pretreatments (Montecchio et al., 2017a), different microbial pathways such as syntrophic acetate oxidation (Capson Tojo et al., 2016), codigestion with activated sludge (Xie et al., 2017; Montecchio et al., 2019), and two-stage configuration (Parra-Orobio et al., 2020).

The model allows for the determination of parameters, such as the biodegradable fraction, which cannot be determined by classic biochemical analysis. In this view, a proper modelling provides information which could not be determined otherwise. For example, Esposito et al. (2011) modified the original ADM1 to determine the relationship between FW physical characteristics, such as the particles size, and the hydrolysis rate.

Nevertheless, biogas production from FW presents additional technological issues. For example, the rapid hydrolysis and acidogenic fermentation steps of FW could result in inhibition factors affecting the overall process stability (Braguglia et al., 2018). The promptly degradable carbohydrate fraction of FW, in fact, selects stable and active fermentative populations, in which Firmicutes and Bacteroidetes are often the most abundant acidogens, as described in Tonanzi et al. (2018; Tonanzi et al., 2020), rather than stable methanogenic populations.

Transforming this problem into an opportunity, a part of the organic waste could be diverted to volatile fatty acids (VFAs) fermentation increasing the diversification of resource recovery. The chemical energy could be recovered in the form of VFAs, which are intermediates produced along the biogas fermentation, being the economic value of VFAs also higher than biogas.

In the context of circular valorization of organic waste, multi-purpose and versatile schemes, integrating thermal and biochemical conversion processes, will play a key role. In such logic, a novel biorefinery platform integrating a thermal enhanced solid-liquid separation unit has been here designed to unlock food waste potential through anaerobic bioconversion processes, by extracting decreasing added-value substances from the liquid phase of a real FW, and converting only the final solid residues into energy.

The potential of such platform to produce short- and medium-chain VFAs by treating the liquid fraction of FW was already described in Gianico et al. (2021) and Gazzola et al. (2022).

This work intends to explore in depth the anaerobic downstream processing of the residual solid streams obtained after a thermally enhanced solid–liquid separation process carried out on raw food waste. For this purpose, particular attention was paid to biomethane yields, biodegradability, microbial dynamics, methanogenic pathways, and process stability. Calibration, validation and sensitivity analysis of the Anaerobic Digestion Model n°1 have been moreover performed on these particular feedstocks to assess the impact of the most relevant kinetic and stoichiometric parameters on semi-continuous mesophilic anaerobic digestion performances.

## 2 Materials and methods

### 2.1 Substrate and inoculum

FW was collected from the cafeteria of the research area “Roma 1” of the National Research Council. As described in Gianico et al. (2021), the cafeteria produced around 200 kg of FW per week, which consisted of mixed raw and cooked food such as fruit and vegetable peelings (70%), cheese (15%), bread and pasta (15%). FW was manually selected to maintain a fixed composition, then it was shredded by a lab-scale knife mill (particle size <1 cm), and subsequently stored at −20°C. For the start-up of anaerobic digestion tests, an inoculum originated from a full-scale sludge anaerobic digester was collected and acclimated by feeding FW daily.

To assess the anaerobic biodegradability of the substrates, Biomethane Potential (BMP) tests were carried out by using the Automatic Methane Potential Test System (AMPTS-II, Bioprocess Control, Sweden). AMPTS-II, consisted of 15 parallel batch reactors of 400 mL of working volume. Each reactor was mechanically stirred (mixing time: 90 s on/120 s off, speed adjustment: 86%). The CO_2_-fixing unit vials were filled with 80 mL of 3N NaOH each, thus allowing the absorption of carbon dioxide and sulphuric acid. As described in Gallipoli et al. (2020), methane flow rate and volume was continuously measured online using automated data logging system with normalization of gas measurement at T = 0°C and *p* = 1 atm. Each reactor was partially filled with inoculum and substrate, according to a substrate/inoculum (S/I) ratio of 0.5, on a VS basis. A blank test was carried out by filling a reactor with inoculum and water.

### 2.2 Experimental set-up

**FIGURE 1:**
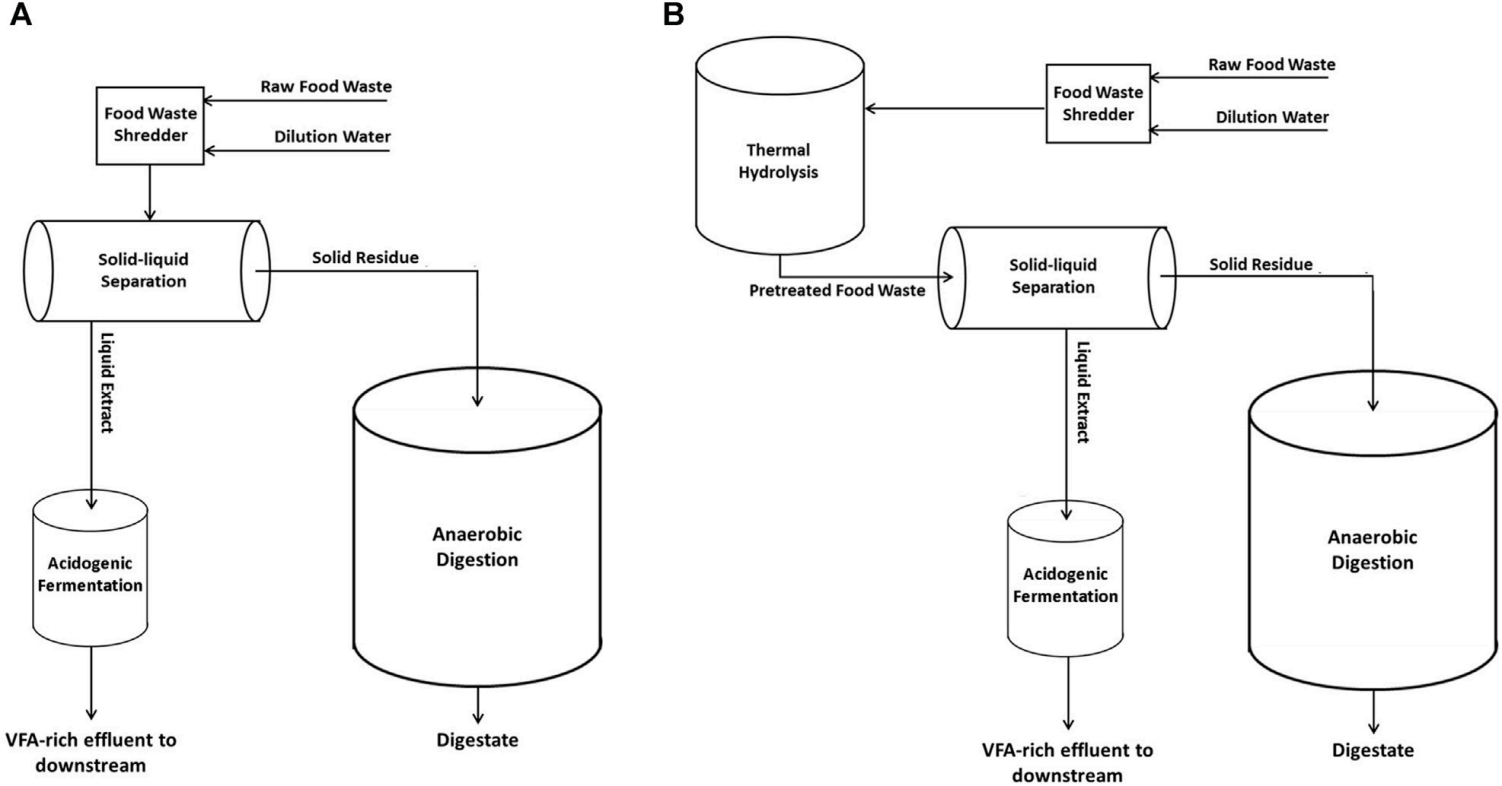
Experimental set-up for solid-liquid phase separation of raw FW **(**panel **A)** and thermal pretreated FW **(**panel **B)**.

The experimental scheme is illustrated in Figure 1. As described in panel A, raw FW was diluted with water (1/1 w/w) and then centrifuged via a lab-scale centrifuge Rotanta 460 (Hettich, Germany) operating at 4,600 rpm for 10 min to separate the liquid extracts and the residues (cake). The obtained solid raw residue was treated through semi-continuous mesophilic (37°C) anaerobic digestion (Exp1 and Exp3). Another diluted FW sample (1/1 w/w) was thermally pretreated for 20 min (by autoclave Laboklav 25b, SHP Steriltechnik AG, Germany) at T = 134°C and *p* = 3.2 bar. Afterwards, as described in Figure 1 - panel B, the pretreated FW was centrifuged and the pretreated solid residue was used as feedstock for the anaerobic digestion tests (Exp2 and Exp4).

### 2.3 Reactor configuration and operation parameters in semi-continuous mode

Anaerobic digestion (AD) tests were carried out on the FW solid residues deriving from the centrifugation unit, using 10 L stainless steel continuous stirred-tank (CSTR) bioreactors (Bioprocess Control, Sweden). As reported in Supplementary Table S1, the first two semi-continuous tests were operated at OLR = 1.6 ± 0.1 gVS L^-1^d^-1^ and HRT = 20 d, by feeding raw residue (Exp1) and thermal pretreated residue (Exp2), respectively. The second two semi-continuous tests were operated at OLR = 1.6 ± 0.1 gVS L^-1^d^-1^ and HRT = 40 d, by feeding raw residue (Exp3) and thermal pretreated residue (Exp4), respectively. The reactors were fed manually 5 times a week (Monday to Friday).

As described in Gianico et al. (2021), the biogas collected from each reactor was sent through a CO_2_ trap (filled with 3M NaOH solution) and then through a methane/hydrogen detection unit (µFlow gas flow meter, Bioprocess Control, Sweden) equipped with temperature and pressure compensation for the normalization of gas flow rate and volume measurement at T = 0°C and *p* = 1 atm. Methane production was measured continuously, also during the weekends.

### 2.4 Analytical methods

Total (TS) and volatile solids (VS), soluble (CODsol) and total COD (CODtot), total (Ntot) and ammonium nitrogen (NH_4_^+^-N), and total and soluble proteins and carbohydrates were determined according to Tonanzi et al. (2020). The lipid content of FW was evaluated by difference. Total phosphorus was determined in triplicates using the phosphate cell tests by Spectroquant (Merck). Lignin and structural carbohydrates (hemicellulose, starch, and cellulose) were determined according to Pagliaccia et al. (2019).

The pH was measured using a Mettler Toledo InPro combined electrode. Volatile fatty acids (VFA) were analyzed by injecting 1 µL of filtered (0.2 µm) and acidified sample into a Perkin Elmer Auto System Gas-Chromatograph equipped with a flame ionization detector (FID), as described in Montecchio et al. (2017b).

Biogas composition was measured using a PerkinElmer Auto System Gas Chromatograph equipped with a thermal conductivity detector (TCD) as reported in Gianico et al. (2021).

Specific methane production (Eq. 1) was calculated as the ratio between the cumulative methane production and the cumulative VS fed to the reactor.

SMPNm3kgVSfed=CH4 Nm3VSfedkg (1)

To examine the statistical significance of the results, the Student’s t-test was performed using Excel; the level for accepted statistical significance was *p* < 0.05.

### 2.5 Microbiological analysis

As already described in Gazzola et al. (2022), anaerobic digestate samples were collected at different sampling times to perform the analysis of microbial community composition over the reactor operation. A small aliquot (2 mL) was centrifuged at 15,000 rpm for 2 min, the resulting pellet was immediately stored at −20°C and used for DNA extraction with DNeasy PowerSoil Pro Kit (QIAGEN - Germantown, MD) following the manufacturer’s instructions. After checked the quality (1.6 < A260/280 < 1.8 and A260/230 > 2) with a Nanodrop 3,300 (Thermo Scientific, Italy), the genomic DNA was used for the high-throughput sequencing of 16S rRNA gene. The V1-V3 region of bacterial 16S rRNA gene (27F 5′- AGA​GTT​TGA​TCC​TGG​CTC​AG-3; 534R 5-ATT​ACC​GCG​GCT​GCT​GG-3) was sequenced following library preparation and protocol described in Crognale et al. (2019). The samples were paired end sequenced (2 × 301bp) on a MiSeq platform (Illumina) using a MiSeq Reagent kit v3, 600 cycles (Illumina, United States) following the standard guidelines for preparing and loading samples, with 20% of Phix control library. Bioinformatic analysis were performed using QIIME2 v. 2018.2 (Bolyen et al., 2019) as described in Crognale et al. (2021). The Dataset is available through the Sequence Read Archive (SRA) under accession PRJNA1052454.

### 2.6 Model

#### 2.6.1 Model description

The ADM1 is a stoichiometric and kinetic model, accounting for 27 variables (24 in the liquid phase and 3 in the gas phase) and 19 biochemical process. The model is intended to represent a completely mixed reactor, such as a Continuous Stirred Reactor (CSTR), which is a traditional configuration for the digestion process. The ADM1 is based on the mass continuity equation (Eq. 2) reported below (Rosen and Jeppson, 2006):

$$\frac{{dC}_{i}}{dt}=\frac{Q}{Vol}∙\left(C_{INPUT,i}-\;C_{i}\right)+\sum_{j=1}^{19}\nu_{i,j}\rho_{j}$$ (2)

where C is the variable concentration, C_INPUT_ is the variable input concentration, Q is the flow rate, Vol is the reactor volume, ν is the stoichiometric coefficients and ρ is the process rate, as defined by the Petersen Matrix reported in Rosen and Jeppson (2006).

Overall, 12 soluble variables (sugars, amino acids, long chained fatty acids, valerate, propionate, butyrate, acetate, soluble inerts, methane, hydrogen, inorganic carbon and nitrogen) and 12 particulate variables (carbohydrates, proteins, lipids, particulate inerts, total particulates and 7 typologies of microorganisms) are included in the model. The processes accounted for in the ADM1 are disintegration/hydrolysis of the particulate compounds, fermentation (which is splitted into acidogenesis and acetogenesis), methanogenesis (hydrogenotrophic and acetoclastic) and dead microorganisms decay. Inhibition factors mostly affect the kinetics of fermentation and acetoclastic methanogenesis and are based on the concentration of hydrogen, free ammonia nitrogen and pH; the latter is calculated through the electroneutrality equation. It is also worth observing that the hydrogen-based inhibition factor is intended to mimic the thermodynamic feasibility of the acetogenic pathways (Batstone et al., 2002; Paton amd Rodriguez, 2019). The pH computation was performed following the approach proposed by Montecchio et al. (2017c).

Finally, biogas production is modeled through classic gas-phase equations, which are based on mass transfer coefficients (k_La_).

#### 2.6.2 ADM1 input variables

Modelling a complex and heterogeneous substrate such as FW is quite challenging; in fact, every stock of substrate daily fed into the digester is supposed to be fully characterized in terms of the 24 model variables, which is not possible. It’s important to note that the substrate stock is characterized in terms of VS, whereas the model requires the COD concentration; moreover, the COD/VS ratio is variable and difficult to determine for each stock. For these reasons, the model input was represented by the average COD/VS ratio capable to close the overall mass balance of the experiment in terms of COD. Therefore, for each experiment a constant COD/VS ratio was adopted, as reported in Supplementary Table S1.

Each substrate stock daily fed into the digester was characterized in terms of COD and the total COD fed was determined through the COD/VS ratio characteristic of the experiment.

To build up the ADM1 input, a single unit of COD - representing the average substrate compositions - was characterized in terms of total, soluble and particulate (calculated by difference) COD. Particulate COD was then represented by the Xc variable which was splitted into Xpr, Xch and Xli (through the ADM1 stoichiometric coefficients fch, fpr and fli), whereas soluble COD was allocated into Ssu, Saa and Sfa.

#### 2.6.3 Sensitivity analysis and calibration

Sensitivity analysis is aimed at detecting the most sensitive kinetic parameters, namely, the parameters whose variation mostly affects the model outcome (which in the case of AD is methane production). Sensitivity Analysis was performed through the procedure described in Montecchio et al. (2017a); essentially, simulations were run with fluctuating values representing the possible range of each given parameter. The parameters affecting methane yield to the highest extent were detected as the most sensitive ones. In general, these parameters can vary in each configuration and depend on the substrate composition and the operational parameters (Capson-Tojo et al., 2016). However, in case of digesters run at high HRTs, such as those considered in this paper, the most sensitive parameters are the disintegration constant and the inert fraction (Kdis and fxi/fsi in the ADM1 nomenclature). The former is the bottleneck of the disintegration/hydrolysis step, whereas the latter represent the inert organic fraction, that is, the fraction which is not subject to the hydrolytic bacteria attack and therefore does not enter the anaerobic process.

Once the sensitive parameters have been detected, their value has to be estimated for each configuration; this was achieved through an iterative process referred to as calibration.

Calibration was carried out by performing several simulations, with variable values of these parameters, and was based on the comparison of the simulated results with the experimental ones. Calibration outcomes were expressed through an objective functions, which represented the difference between the simulations and the experiments values for a given variable, referred to as the focus variable. In this case, methane was selected as the focused variable. Four objective functions (Eqs 3–6) were selected for this calibration, that are, the Nash-Sutcliffe Efficiency coefficient (NE), the Modeling Efficiency (ME), the Index of Agreement (IoA), Least Square Error (LSE); these functions were calculated as (Koch et al., 2010; Panico et al., 2014).

$$\text{NE}=1-\frac{\sum_{i=1}^{n}\left|S_{obs}^{i}-\;S_{sim}^{i}\right|}{\sum_{i=1}^{n}\left|S_{obs}^{i}-\bar{S_{obs}^{i}}\;\right|}$$ (3)

$$ME=1-\;\frac{\sum_{i=1}^{n}{\left(S_{obs}^{i}-S_{sim}^{i}\right)}^{2}}{\sum_{i=1}^{n}{\left(S_{obs}^{i}-\;\bar{S_{sim}^{i}}\right)}^{2}}$$ (4)

$$IoA=1-\;\frac{\sum_{i=1}^{n}{\left(S_{sim}^{i}-\;S_{obs}^{i}\right)}^{2}}{\sum_{i=1}^{n}{\left(\left|S_{sim}^{i}-\bar{S_{sim}^{i}}\right|+\left|S_{obs}^{i}-\;\bar{S_{sim}^{i}}\right|\;\right)}^{2}}$$ (5)

$$LSE=\frac{\sqrt{\sum_{i=1}^{n}{\left(S_{obs}^{i}-S_{sim}^{i}\right)}^{2}}}{n}$$ (6)

Where:

- S_obs_ is the observed daily methane yield,
- S_sim_ is the simulated daily methane yield,
- $\bar{S_{obs}^{i}}$ is the observed average methane yield
- $\bar{S_{sim}^{i}}$ is the simulated average methane yield
- n is the number of days.

Given the intrinsic heterogeneity and variability of FW, which made almost impossible to select a representative stock of substrate with sufficient accuracy, calibration was performed on long-lasting semicontinuous experiments which, in this view, are far more reliable than BMP tests.

## 3 Results and discussion

Raw FW, rich of water because of the abundance of vegetable scraps and fruit peelings, underwent a solid/liquid separation providing a solid residue characterized by high concentration of solids mainly composed by carbohydrates in the form of starch, proteins and lignocellulosic components. The residue deriving from the thermal pretreated FW, on the contrary, contained noticeably less solids and less organic substance (Table 1); in fact, by increasing the efficacy of extraction of organics into the liquid phase, the thermal hydrolysis affected the total COD content of the pretreated solid residue, which resulted ∼30% lower with respect to the raw one (Table 1). In particular, the pretreated residue was depleted by a significant fraction of complex carbohydrates, as starch, converted in soluble carbohydrates and transferred into the liquid phase. Soluble bio-available COD was 15% and 26% of the total COD for the raw and the pretreated residue, respectively.

**TABLE 1:** Characterization of the raw FW (before pretreatments), and characterization of the solid residues and liquid extracts obtained after phase separation of raw and thermal pretreated FW.

	Raw FW	Raw FW after centrifugation		Pretreated FW after centrifugation	
Parameter (g/kgFW)	*Before Pretreatments*	*Solid Residue*	*Liquid Extract*	*Solid Residue*	*Liquid Extract*
TS	220 ± 16	185 ± 15	35 ± 2	116 ± 8	104 ± 8
VS	209 ± 15	178 ± 14	31 ± 2	112 ± 8	97 ± 7
Total COD	249 ± 21	212 ± 20	37 ± 4	151 ± 14	98 ± 10
Total Carbohydrates	119 ± 10	93 ± 9	26 ± 3	63 ± 6	56 ± 6
Total Proteins	35 ± 2	27 ± 2	8 ± 1	19 ± 2	16 ± 1
Total Lipids	8 ± 1	8 ± 1	0	8 ± 1	0
Starch	63 ± 7	60 ± 6	3 ± 1	31 ± 3	7 ± 1
Ligno-cellulosic matrix	40 ± 3	39 ± 4	1 ± 1	30 ± 3	9 ± 1
Soluble COD	69 ± 6	32 ± 3	36 ± 4	40 ± 4	71 ± 7
Soluble Carbohydrates	41 ± 3	19 ± 2	22 ± 2	20 ± 2	46 ± 5
Soluble Proteins	16 ± 2	8 ± 1	8 ± 1	11 ± 1	16 ± 2

To assess the anaerobic biodegradability of both residues, a preliminary BMP test was performed. The BMP test was conducted at mesophilic conditions (37°C) for 80 days. Supplementary Table S3 shows the characterization of the batch reactors before and after the digestion tests. The obtained methane conversion rates were 0.48 ± 0.02 Nm^3^/kgVS_fed_ both for raw and pretreated residues, thus demonstrating that thermal pretreatment did not affect the extent of biodegradation of the FW residues in long-term batch digestion tests. It is worth noting that the BMP test carried out on the same raw FW without any pretreatment or phase separation showed lower conversion rates of 0.38 ± 0.01 Nm^3^/kgVS_fed_ (Gallipoli et al., 2020); this is due to the presence of a higher soluble and rapidly fermentable organic fraction which hampers methanogenesis.

### 3.1 Anaerobic digestion of solid residues: effect of thermal pretreatment on methane production (HRT = 20d)

**FIGURE 2:**
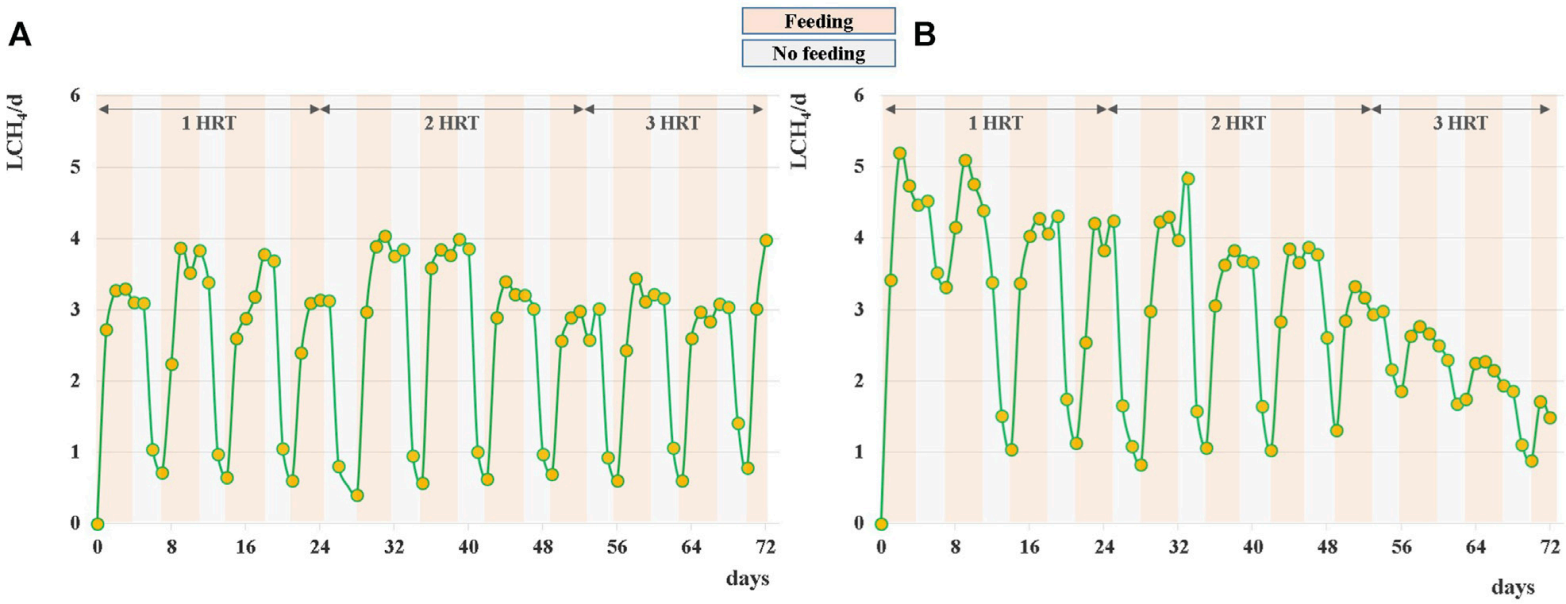
Daily methane production obtained during Exp1 **(A)** and Exp2 **(B)** at HRT = 20d.

**FIGURE 3:**
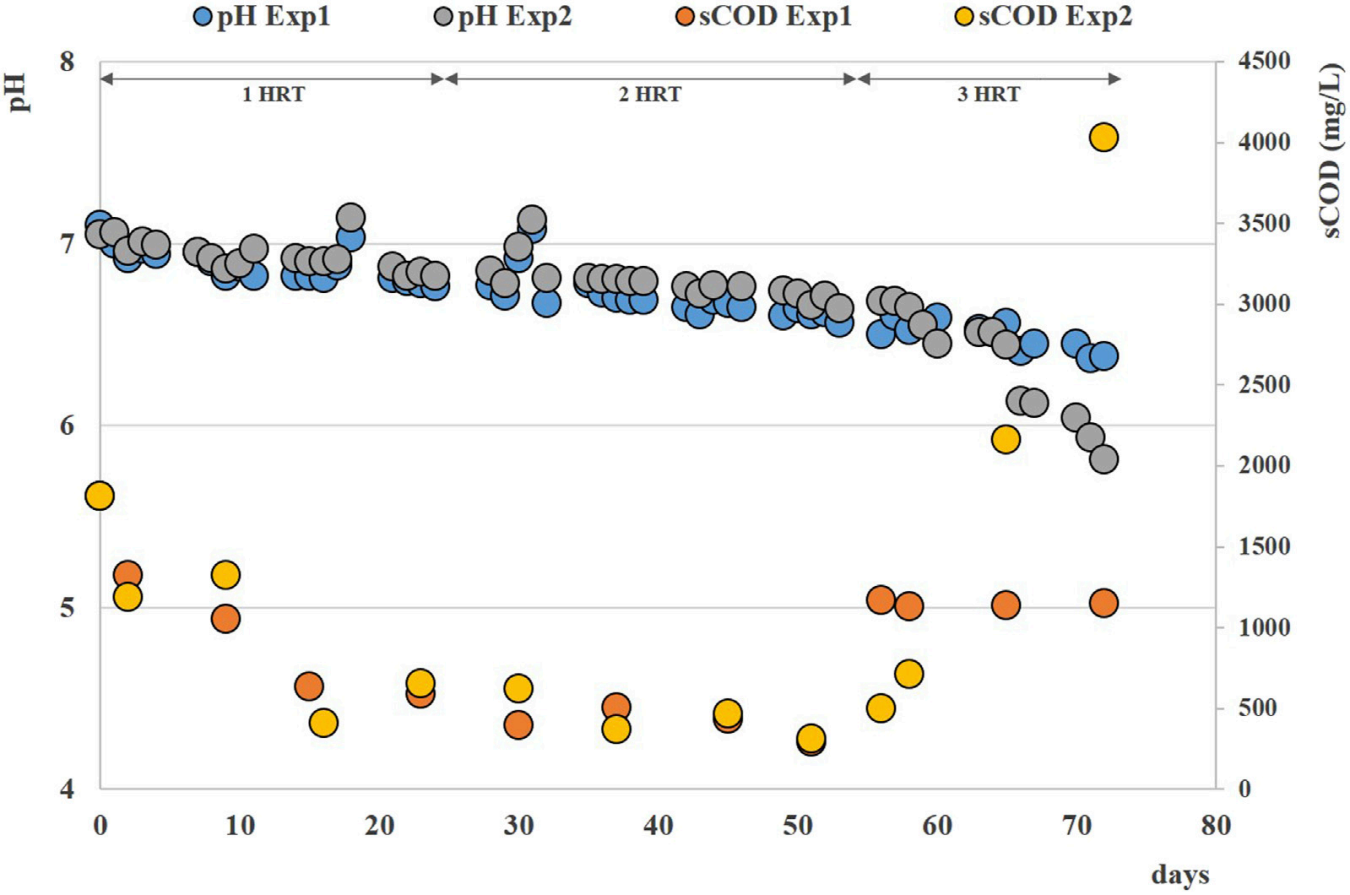
Soluble COD and pH patterns obtained during Exp1 and Exp2 at HRT = 20 d.

During the anaerobic digestion tests carried out at HRT = 20 d in semi-continuous mode, the pretreated residue evidenced significantly higher methane conversion rates (*p* < 0.05), with a specific production of 0.33 ± 0.01 Nm^3^CH_4_ kg^-1^VS_fed_ against 0.29 ± 0.01 Nm^3^CH4 kg^-1^VS_fed_ of the untreated one during the stable period that lasted approximately 2 HRTs. Nevertheless, long-term operation evidenced unstable conditions; in fact, after 50 days, soluble COD and volatile fatty acids accumulated, and both pH and methane dropped down especially for the pretreated substrate (Figure 2B; Figure 3; Supplementary Figure S1B). The depletion of the soluble bio-available content due to liquid/solid separation avoided rapid acidification and methane drop typically observed with this FW operating at comparable short HRT (Tonanzi et al., 2018; 2020), but did not impede the collapse of the AD system fed with solid residues. An initial stability of both systems was reflected by the soluble COD patterns: the high initial soluble organic content of both reactors, due to the previous acclimation phase with FW, was in fact efficiently removed and converted to methane, thus evidencing the healthy status of the methanogenic biomass (Figure 3). Over the stable period, the soluble COD level settled at 0.5 ± 0.1 g L^-1^ in both reactors, without VFAs accumulation. Constant pH values of 6.7 ± 0.1 and 6.8 ± 0.1 were recorded for both Exp1 and Exp2, due probably to the buffering capacity of both systems, as indicated by the soluble ammonia Nitrogen at around 1 g L^-1^ (Figure 3; Table 2).

**TABLE 2:** Characteristics of feeds and digestates during the sable states of Exp1 and Exp2 (HRT = 20d).

	Exp1		Exp2	
Parameter	Feed	Digestate	Feed	Digestate
TS (g kg^-1^)	32.2 ± 1.8	11.8 ± 3.5	32.1 ± 2.2	12.5 ± 2.9
VS (g kg^-1^)	31.1 ± 1.7	9.0 ± 2.2	31.1 ± 2.1	9.5 ± 1.6
CODtot (g L^-1^)	37.3 ± 3.5	11.4 ± 1.4	40.4 ± 5.0	12.4 ± 1.2
CODsol (g L^-1^)	5.3 ± 0.9	0.5 ± 0.1	2.8 ± 0.6	0.5 ± 0.1
Total Nitrogen (%TS)	3.4 ± 0.4	15.3 ± 3.5	3.8 ± 0.7	15.3 ± 2.6
Total Phosphorus (%TS)	0.31 ± 0.03	1.66 ± 0.65	0.25 ± 0.04	1.31 ± 0.55
Ammonia (gNH_4_^+^-N L^-1^)	n.d	1.02 ± 0.18	n.d	1.09 ± 0.24
VFA (gCOD L^-1^)	n.d	0.21 ± 0.02	n.d	0.22 ± 0.03

The organics removal (in terms of volatile solids reduction) increased over time, and during the last HRT the average value was around 73% ± 5% for both AD tests, in accordance with previous findings reported elsewhere (Yi et al., 2014; Tonanzi et al., 2018). After 50 days (during the third HRT) the situation changed, soluble COD started accumulating because of the generation of VFAs not balanced by their consumption rate (See Supplementary Figure S1A, B), and soluble COD reached 4 g/L at the end of the operation. Buffering capacity failed and pH dropped down to 5.8 in the reactor fed with pretreated residue (Figure 3).

Compared to literature values, the specific methane production of these residues during stable state were slightly higher than those observed by Valentino et al. (2019), who recovered 0.25 and 0.29 Nm^3^CH_4_ kg^-1^VS_fed_ (in mesophilic and thermophilic regimen, respectively) from the AD step of a combined acidic fermentation and anaerobic digestion treatment, feeding the reactor with the solid-rich fraction of a pre-fermented mixture of waste activated sludge (WAS) and municipal organic solid waste (OFMSW).

Daily average methane production was 2.6 ± 0.1 L and 3.0 ± 0.2 L for the raw and pretreated residue, respectively, during the stable-state period (Figure 2).

Interestingly, in both systems the methane production continued even during the weekend (when feeding was suspended), differently from what was observed by digesting raw FW at the same organic load (Tonanzi et al., 2018). As evidenced in Figure 2, the methane production during the no-feeding days was always higher (+20%) for the pretreated residue. This behavior may be ascribed to the weakening of the lignocellulosic matrix and the increase of the specific surface and matrix exposure area induced by the thermal pretreatment (Pagliaccia et al., 2019; Gallipoli et al., 2020; Freitas et al., 2023), that promoted the availability of a slowly-biodegradable fraction for the anaerobic microorganisms in Exp2.

**FIGURE 4:**
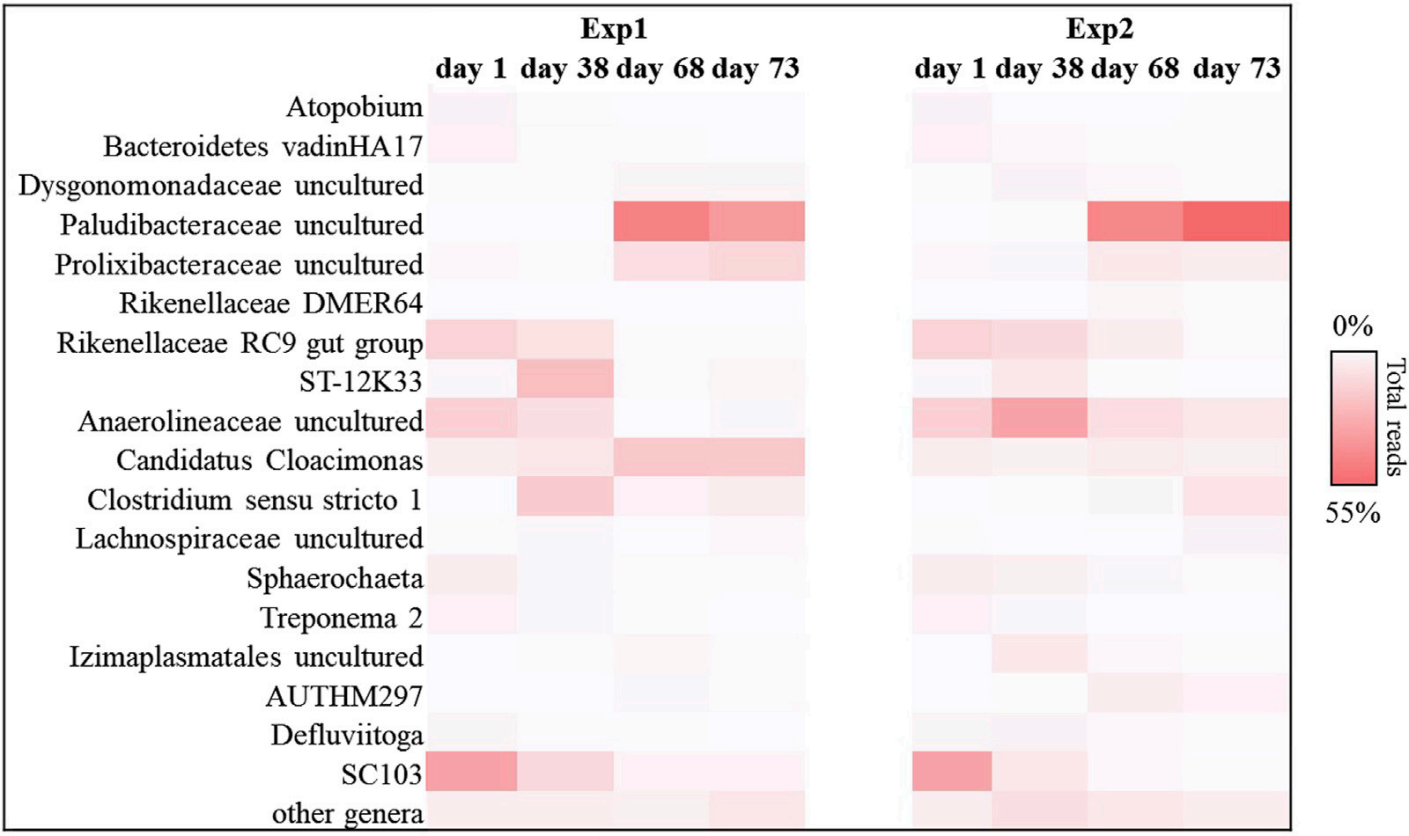
Reads frequency heat-map of bacterial communities at genus level in Exp1 and Exp2 (HRT = 20d). Only taxonomy groups with ≥2% abundance in at least one sample are shown. The color intensity shows the relative abundance of the sequences (min 0%, white; max 55%, red).

The bacterial and archaeal composition and dynamics were investigated during all duration of the studied tests. High throughput 16 S rRNA gene sequencing revealed that the main bacterial phyla observed during Exp1 and Exp2 were *Bacteroidetes*, *Chloroflexi*, *Cloacimonetes*, *Firmicutes*, and *Thermotogae*, which represented between 85% and 90% of the total reads in all the samples sequenced (Supplementary Figure S2). However, the selected HRT of 20 days, together with the complex organic content of the residues, resulted to favour *Bacteroidetes*, hydrolitic and narrow fermenters, in particular for Exp2, in which a greater selection was observed (Supplementary Figure S2). Most of the sequences found in both tests belonged to the *Paludibacteraceae* family, which after 2 HRTs were selected reaching over 55% of the total sequences in Exp2 (Figure 4). This family consist of strictly anaerobic chemoorganotrophic bacteria able to ferment various sugars which can be used as carbon source with acetate and propionate as major fermentation end-products (Ueki et al., 2006). The abundance of these organisms in the acidification step of Exp2 (after 2 HRT, see Figure 4; Supplementary Figure S1B) mirrored the VFA production detected within the reactor.

**FIGURE 5:**
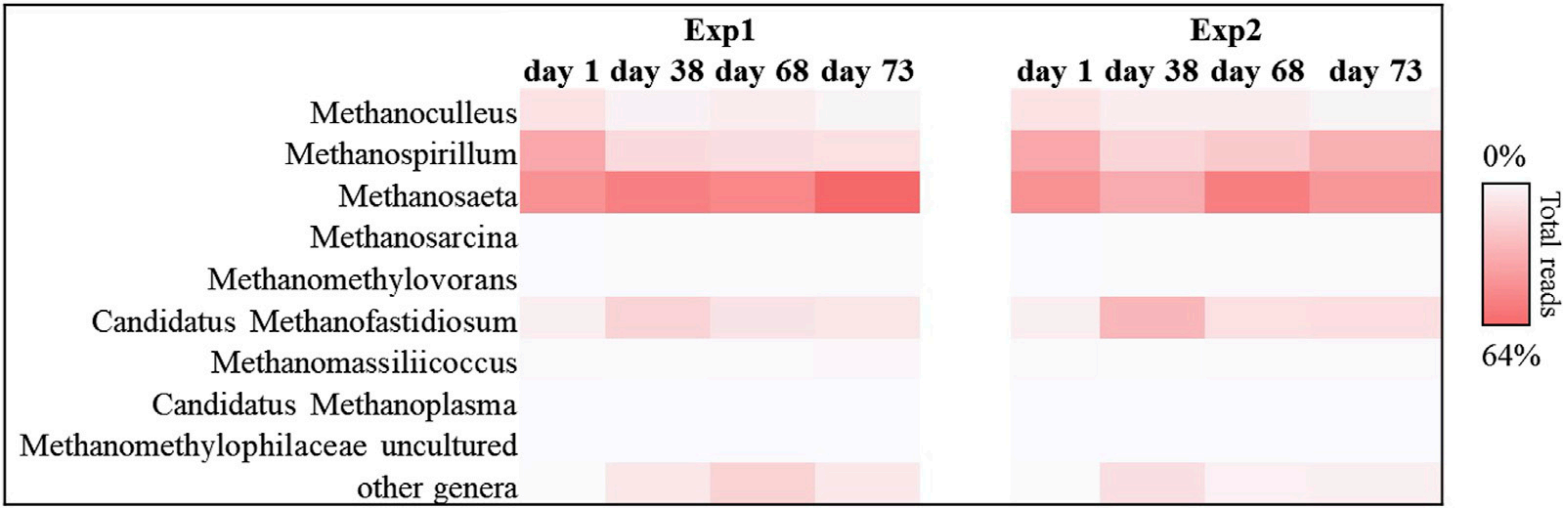
Sequences frequency heat-map of Euryarcheota archaeal communities at genus level in in Exp1 and Exp2 (HRT = 20d). Only genera with ≥1% reads abundance in at least one sample are shown. The color intensity shows the relative abundance (min 0%, white; max 64%, red).

This trend was also confirmed by the results obtained for the methanogenic component. In particular, for each sample sequenced, Euryarchaeota phylum covered about 99% of the total reads investigated. In detail, the methanogenic archaeal relative abundance (Figure 5) showed that for Exp1 the methanogenesis occurred through a mainly acetoclastic pathway. This result is in line with previous studies reporting the transformation of acetate to methane by acetoclastic methanogens as a major pathway in mesophilic anaerobic digestion of FW (Zamanzadhe et al., 2016; Tonanzi et al., 2018). The number of reads affiliated with the genus *Methanosaeta* increased from 36% to 64% of the total sequences, from the start of the experiment to the end (Figure 5), mirroring the healthy state of the methanogenic biomass involved in this experiment. Conversely, acetoclastic and hydrogenotrophic metabolism coexisted in Exp2 (Figure 5). In this case, the sequences belonging to the *Methanosaeta* genus were stable overall throughout the test (44% ± 8% of the total reads), however, a strong central hydrogenotrophic microbiome was established in the reactor. In Figure 5 clearly shows how the genus *Methanospirillum* had a predominant role in this system, reaching up to 30% of the total reads, as well as the *Candidatus Methanofastidiosum* genus, for which an increase of up to 70% was observed compared to the initial reads found. The increase of these hydrogenotrophic taxa suggested the accumulation of hydrogen in the reactor, thus highlighting an imbalance which led to the failure of methanogenesis: the increased bioavailability of the organic load in Exp2 led to the acceleration of the hydrolysis-acidification process, selecting the hydrogenotrophic methanogens which, however, were unable to consume all the hydrogen produced thus leading to the accumulation of reduced metabolites, such as VFAs (Hagen et al., 2014).

### 3.2 Anaerobic digestion of solid residues: effect of thermal pretreatment on methane production (HRT = 40d)

**FIGURE 6:**
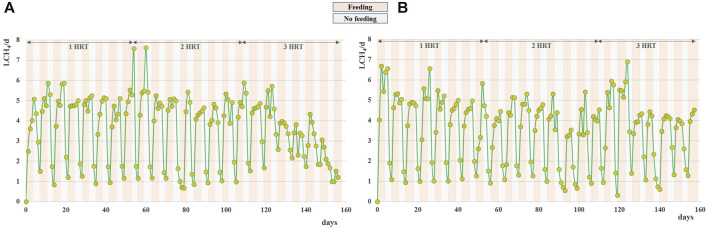
Daily methane production obtained during Exp3 **(A)** and Exp4 **(B)** at HRT = 40 days.

The results obtained from Exp1 and Exp2 carried out at HRT of 20 days suggested to increase the retention time for maximizing the conversion potential of the substrate. For this reason Exp3 and Exp4 were carried out in semi-continuous mode, operating the digesters at HRT of 40 days. As expected, both tests evidenced similar methane conversion rates, with a specific production of 0.33 ± 0.01 Nm^3^CH_4_ kg^-1^VS_fed_, higher with respect to the one obtained at HRT = 20d for the raw residue. Stable digestion processes have been observed since the start-up of both AD tests and maintained for almost 3 HRTs (up to ∼120 days), with an average daily methane production of 3.6 ± 0.2 L for both the raw and pretreated residue, no-feeding days included (Figures 6A,B).

**FIGURE 7:**
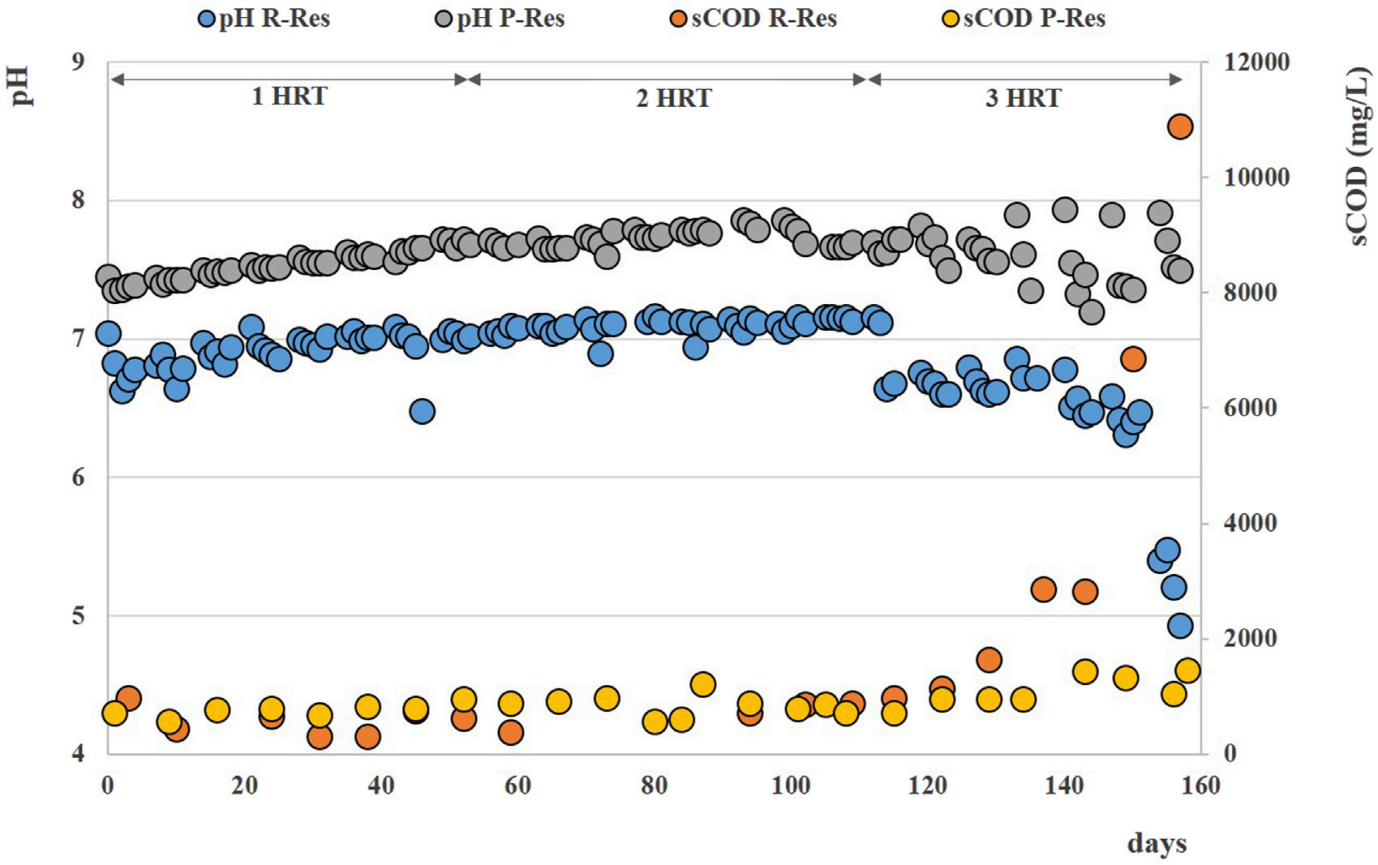
Soluble COD and pH patterns obtained during Exp3 and Exp4 at HRT = 40 days.

Over the stable period, the soluble COD remained always below 1 g L^-1^ in both reactors, without significant accumulation of VFAs (Table 3); constant pH values of 7.1 ± 0.1 were recorded for Exp3, while an increasing trend of pH was observed for Exp4, reaching values of 7.6 ± 0.3 at the end of the test (Figure 7).

**TABLE 3:** Characteristics of feeds and digestates during the sable state of Exp3 and Exp4 (HRT = 40 d).

	Exp3		Exp4	
Parameter	Feed	Digestate	Feed	Digestate
TS (g kg^-1^)	67.4 ± 1.8	15.7 ± 1.8	63.8 ± 3.5	21.2 ± 2.9
VS (g kg^-1^)	64.2 ± 1.7	12.1 ± 1.7	61.6 ± 4.5	16.2 ± 2.8
CODtot (g L^-1^)	76.2 ± 7.9	14.3 ± 1.9	80.1 ± 6.9	19.5 ± 2.7
CODsol (g L^-1^)	14.1 ± 2.2	0.8 ± 0.3	15.7 ± 2.3	0.8 ± 0.2
Total Nitrogen (%TS)	4.1 ± 0.3	11.5 ± 1.3	4.4 ± 0.2	10.1 ± 1.2
Total Phosphorus (%TS)	0.4 ± 0.1	2.9 ± 0.4	0.5 ± 0.1	3.2 ± 0.3
Ammonia (gNH_4_^+^-N L^-1^)	n.d	0.91 ± 0.18	n.d	0.99 ± 0.10
VFA (gCOD L^-1^)	n.d	0.29 ± 0.04	n.d	0.27 ± 0.05

The volatile solids reduction (%) during stable state was around 81% ± 2% for Exp3 and 75% ± 5% for Exp4. For the tests conducted at HRT 40 days, in both digesters the methane evolution proceeded even during the weekend (when feeding was suspended). However, unlike previous tests at HRT 20 days, less differences in methane production were observed during Saturday and Sunday between untreated and pretreated FW residues (Figure 6). This result is ascribed to the longer hydraulic retention time applied that favoured the degradation of the slowly-degradable fraction, also for the untreated residue.

After 120 days (during the fourth HRT), VFAs started accumulating in the reactors (See Supplementary Figure S1C, D) and soluble COD reached ∼11 g/L at the end of the operation of Exp3, with final pH values lower than 5 (Figure 7).

Nevertheless, neither the retention time at 20 days, nor that at 40 days were found to be sufficient to exploit all the methane potential of the food waste residues. In fact, in terms of methane conversion rates during stable state (Nm^3^CH_4_ kg^-1^VS_fed_), the semi-continuous tests are always characterized by lower performances compared to the BMP tests. This because the hydrolysis of the slowly degradable COD fraction of the food waste residues is rate limiting for the digestion process.

The thermal pretreatment, by weakening the lignocellulosic fraction, was partially effective in the reduction of the slow degradable organics, thus leading an improvement of the digestion rates. On the contrary, no effects of pretreatment were observed on the extent of food waste residue degradability, as demonstrated by the results of BMP tests (same methane potential between R-Res and P-Res).

Moreover, the specific methane production obtained in semi-continuous tests using the pretreated food waste residues (up to 0.33 Nm^3^CH_4_ kg^-1^VS_fed_) resulted also 27% higher with respect to a conventional digestion process carried out on raw food waste without any pretreatment or phase separation (∼0.26 Nm^3^CH_4_ kg^-1^VS_fed_ at HRT = 40 days) (Tonanzi et al., 2021).

The 16 S RNA sequencing analysis was also performed to investigate the biomass dynamics in Exp3 and Exp4. The bacterial composition analysis, revealed that *Actinobacteria*, *Bacteroidetes*, *Firmicutes,* and *Thermotogae* were the main taxa also in the reactors operated at HRT = 40d (between 80% and 90% of the total reads, in each sample investigated; see Supplementary Figure S3). Among these bacteria, *Actinobacteria* phylum was predominant in Exp3 after 3 HRT, when the anaerobic digestion process was unstable (up to 36% of the total sequences at the end of the operation, fold increase of 93%). Members of this phylum have previously been related to poor methane yield in unstable anaerobic digestion processes of FW, as they are mainly acidogenic and promote VFA accumulation (Zhang et al., 2018; Ali et al., 2019; Zhong et al., 2020; Ma et al., 2021). Exp3 was characterized by the increase of reads belonging with *Thermotogae* phylum (up to 95% of increase at the end of the operation with respect to day 1).

**FIGURE 8:**
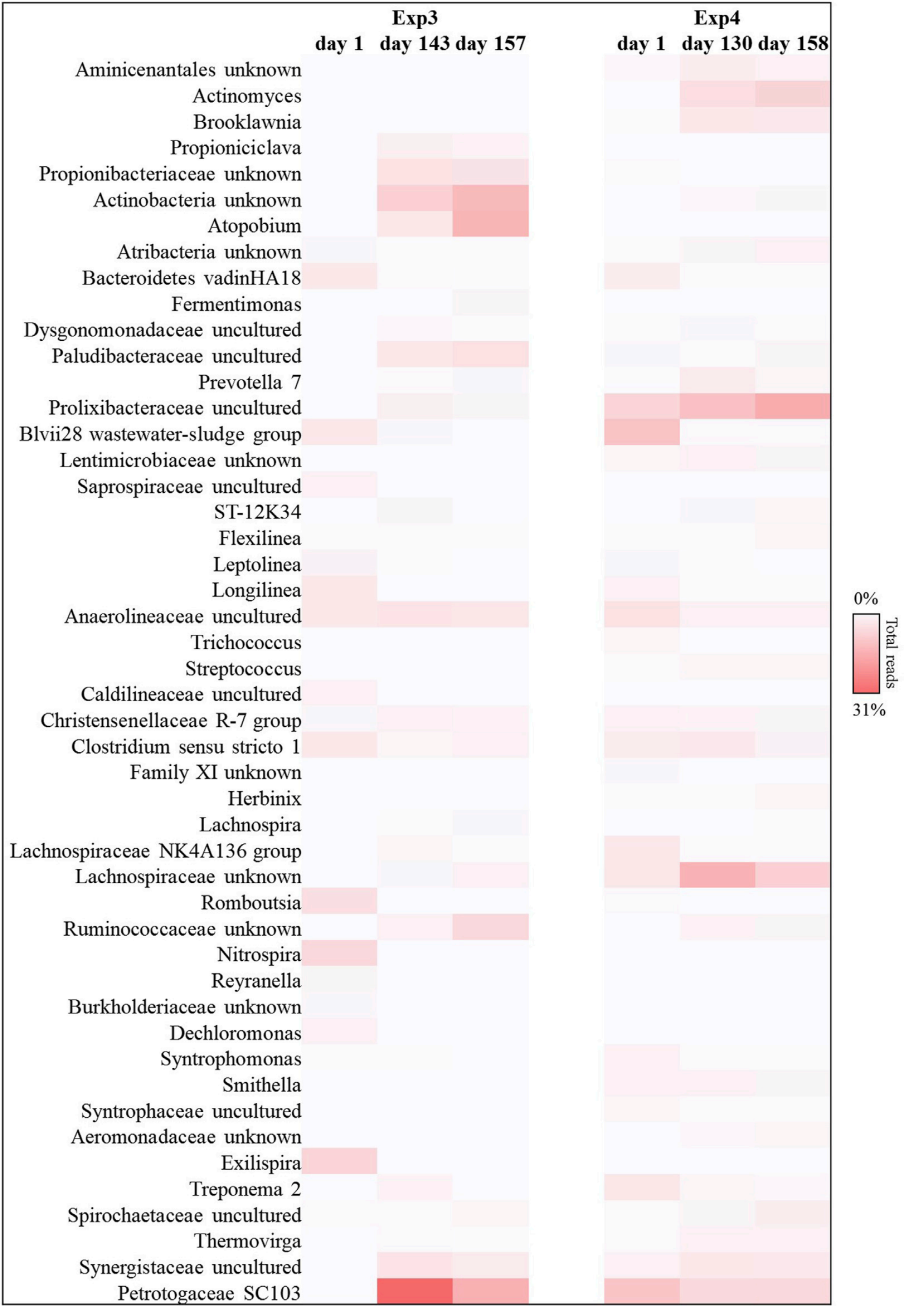
Reads frequency heat-map of bacterial communities at genus level in Exp3 and Exp4 (HRT = 40d). Only taxonomy groups with ≥2% abundance in at least one sample are shown. The color intensity shows the relative abundance of the sequences (min 0%, white; max 31%, red).

In particular, *Petrotogaceae* family represented the main taxa affiliated with *Thermotogae* phylum (Figure 8). The latter group has previously been described in systems where hydrogen production was high because these microorganisms are capable of degrading a wide range of simple and complex carbohydrates, producing high-yield fermentative hydrogen (Frock et al., 2010; Li et al., 2016).

On the other hand, Exp4 was characterized by the presence of an evident hydrolytic core. *Bacteroidetes* and *Firmicutes* phyla represented about 52% ± 3% of the total sequences, overall operation (Supplementary Figure S3). In particular, *Prolixibacteraceae* (*Bacteroidetes*) and *Lachnospiraceae* (*Firmicutes*) families reached up to 16% and 9% of the total reads, respectively, at the end of the operation (Figure 8). The abundance of these hydrolytic taxa in Exp4, compared to the other tests, confirmed the greater capacity of this biomass to use even more complex matrices made bioavailable by the pre-treatment (e.g., lignocellulosic fraction) for the production of methane, probably also due to the higher HRT.

**FIGURE 9:**
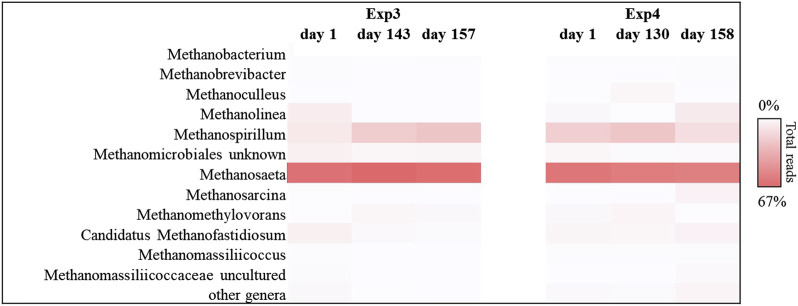
Sequences frequency heat-map of Euryarcheota archaeal communities at genus level in Exp3 and Exp4 (HRT = 40d). Only genera with ≥1% reads abundance in at least one sample are shown. The color intensity shows the relative abundance (min 0%, white; max 67%, red).

By operating the tests at longer HRT (40 days), the acetoclastic metabolism resulted predominant both in the Exp3 and in the Exp4, with high relative abundance of acetoclastic methanogenic biomass, as reported in Figure 9. Most sequences were affiliated with *Metanosaeta* group, which reached up to 65% and 57% of the total reads for Exp3 and Exp4, respectively. The main difference between the two tests was observed in the hydrogenotrophic methanogens portion. *Methanospirillum* genus resulted the most abundant hydrogenotrophic archaea in both reactors (25% and 14% of the total sequences, respectively). Hydrogenotrophic *Methanolinea* and *Candidatus Methanofastidiosum* genera were also present to a lesser extent (0.1% and 8%; 1.5% and 5%, respectively for Exp3 and Exp4. *Methanolinea* and *Methanospirillum* were the major H_2_ scavengers to support syntrophic degrading propionate bacteria and syntrophic oxidizing butyrate, such as *Syntrophobacterales* family, *Pelotomaculum* and *Syntrophomonas* genera (Li et al., 2018). In Exp4 the association of methanogenic hydrogenotrophic biomass, mainly composed by *Methanolinea* and *Methanospirillum,* about 22% of the total reads (Figure 9), with syntrophic bacteria like *Smithella*, *Syntrophobacter* (*Syntrophobacterales* family, about 3% of the total reads, data no shown)*, Syntrophomonas* and *Pelotomaculum* genera (*Clostridium* family, about 1.5% of the total reads, data no shown) probably prevented the acidification of this system.

### 3.3 Modelling

Sensitivity Analysis detected the inert fraction (f_xc_) and the disintegration constant (k_dis_) as the most sensitive parameters (data not shown); this outcome was largely predictable, for the reasons mentioned at par. 2.6.3. Calibration was performed for the part of the experiments where a stable methane production was achieved; in other words, the acidification occurred in the final part of the tests Exp2 and Exp3 was not considered, since other biochemical aspects, which cannot be captured by the original ADM1, took over in the digestion process. This issue was thoroughly discussed in Montecchio et al. (2019).

**FIGURE 10:**
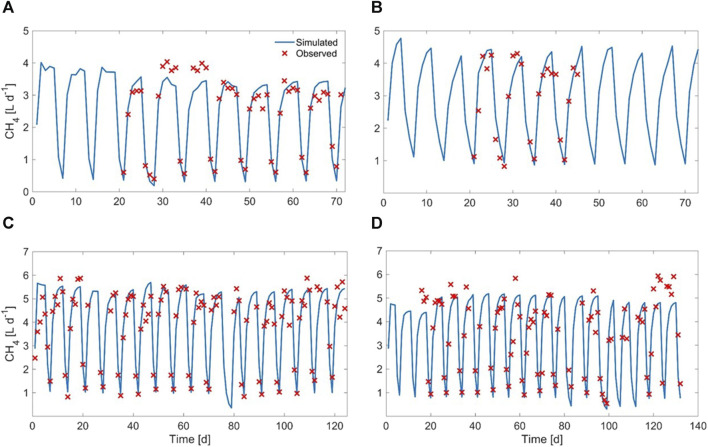
Observed vs. simulated methane yield for **(A)** Exp1, **(B)** Exp2, **(C)** Exp3, and **(D)** Exp4.

**FIGURE 11:**
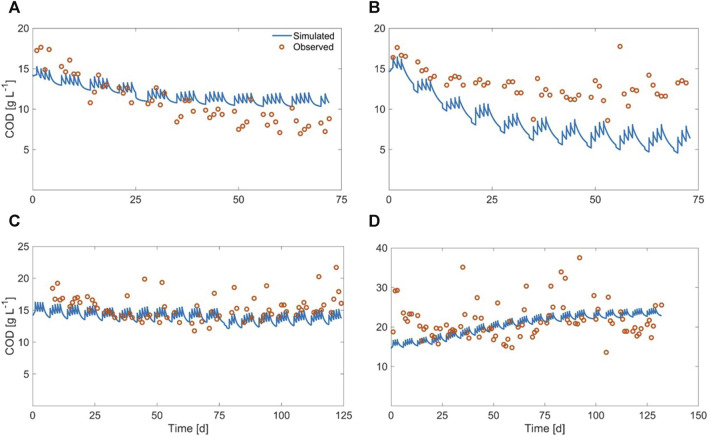
Observed vs. simulated COD output for **(A)** Exp1, **(B)** Exp2, **(C)** Exp3, and **(D)** Exp4.

The graphical comparison between simulated and experimental results, regarding methane yield and COD output, is displayed in Figures 10, 11

The calibration process was based on the calculation of the objective functions ME, NE, IoA and MRS, as reported in par. 2.6.3, with methane selected as the focus variable. For each experiment, the values of the sensitive parameters and the corresponding objective functions are reported in Supplementary Table S3.

It can be observed that the procedure adopted to cope with this issue (described at par. 2.6.3) achieved good results, in spite of the large heterogeneity of the substrate, since the values of all of the objective functions were within acceptable ranges. This was also confirmed by the graphical comparison between simulated and experimental results (regarding methane yield and COD in the digestate) displayed in Figures 10, 11.

Nevertheless, although the model was capable to simulate the average pattern of the methane yield throughout the experiment, it was not possible to detect the minor fluctuations occurring along each feeding week. Most likely, the experimental methane yield fluctuations could be ascribed to the substrate heterogeneity, since different stocks originated from the same sample could still present some physical and chemical differences among one another. The model could not capture these small variations, as it was not possible to adapt the model COD allocation to the minor variabilities of every single stock.

As expected, the selection of the objective function did not affect the values of the calibrated parameters to a significant extent; indeed, both f_xi_ and K_dis_ were nearly independent on the objective function selected (Supplementary Table S3) and were bounded within a very small interval. It’s worth noting that the calibration for Exp2 could possibly not be as reliable as that performed for the other experiments, since only a few data were available before acidification took place.

In general, the inert fraction was low and ranging between 0%–30% of the total COD content; this result is in line with that obtained by Montecchio et al. (2019) for a similar FW substrate and confirms the eligibility of FW solid residues for anaerobic digestion. The disintegration step was also rather fast if compared with other substrates (Esposito et al., 2011; Capson Tojo et al., 2016), since k_dis_ was bounded between 1–1.5 d^-1^, with the exception of the pretreated sample used for Exp2. The latter presented a far lower disintegration constant (k_dis_ = 0.5–0.6 d^-1^), which delayed the substrate digestion, thus resulting in a higher methane production over the weekend, when the reactor was not fed and was operated in batch mode. It’s also worth noting that peculiarity of this FW sample is confirmed by the high COD/VS ratio (1.35), which was slightly higher than that of the other samples.

In general, the variability of the different FW stocks appeared to be the main factor influencing the process efficiency. This was particularly true in the case of the pretreated samples used for Exp2 and Exp4. It was also hard to detect a significant difference between raw and pretreated FW for the selected parameters. Indeed, the main diversity regarded the soluble fraction, as reported in Table 1, which could possibly result in a higher methane production (but also in a higher risk of acidification) especially for experiments conducted at lower HRTs.

### 3.4 Organic mass balance of the biorefinery platform

**FIGURE 12:**
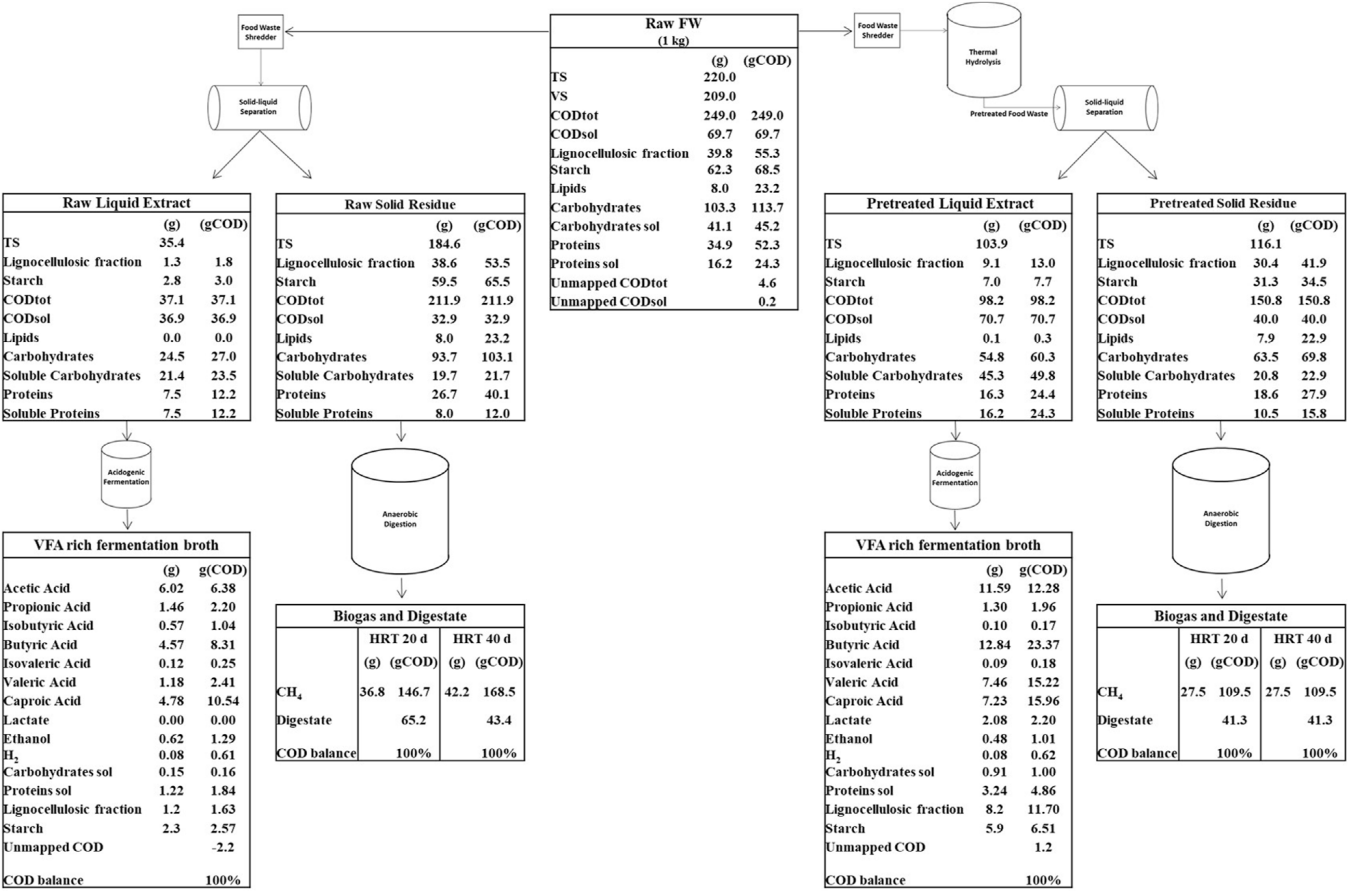
COD mass balance of the Biorefinery platform.

The innovative biorefinery platform designed in this work incorporates multiple unit operations such as thermal hydrolysis, solid-liquid phase separation, fermentation, and anaerobic digestion, orchestrating a cascade of conversion pathways to maximize the extraction of valuable compounds from FW. Although the objective of the paper is to investigate in depth the valorization of the residual solid side streams, a mass balance of the whole biorefinery platform is presented in Figure 12. Starting from the results obtained by lab-scale fermentation tests performed on FW liquid extracts, and by lab-scale anaerobic digestion tests performed on FW solid residues, the overall mass balance was assessed on a COD basis. Such COD balance was determined both for the scenario involving only FW centrifugation and for the scenario involving also thermal pretreatment, with the aim to comprehend the distribution and transformation of organic compounds throughout the various stages of the biorefinery process. To validate the mass balance, COD equivalents of each component were estimated by assuming stoichiometric conversion factors. In detail, the following factors (gCOD g^-1^Compound) were assumed: 1.1 for carbohydrates, 1.5 for proteins, 1.56 for lignin, 2.9 for lipids, 2.09 for ethanol, 1.07 for lactate, 1.07 for acetic acid, 1.51 for propionic acid, 1.82 for butyric and isobutyric acids, 2.04 for valeric and isovaleric acids, and 2.21 for caproic acid. The COD equivalent of hydrogen was assumed as 0.71 gCOD L^-1^H_2_ (H_2_ density = 0.09 g L^-1^); the COD equivalent of methane was assumed as 2.86 gCOD L^-1^CH_4_ (CH_4_ density = 0.72 g L^-1^).

Figure 12 shows the process flow diagram and the related mass balances of the proposed biorefinery platform for valuable by-products generation from 1 kg of FW. According to the experimentally obtained organic mass fractionation of raw FW, the mass transfer into liquid extract and solid residue is given for both scenarios. The conversion of organic matter into VFAs, lactate, ethanol, H_2_ and CH_4_ was calculated assuming the experimental yields obtained during lab-scale acidogenic fermentation tests performed on FW liquid extracts and during digestion tests performed on FW solid residues (Supplementary Table S4).

Thermal pretreatment was effective in transforming part of the complex carbohydrates (in particular starch) into soluble sugars. During the following centrifugation phase, these soluble sugars were then separated and transferred to the liquid stream (Figure 12). Such liquid stream, enriched in organic matter, proved to be an excellent substrate for VFA production via fermentation. As shown by the mass balance, the liquid steam obtained after thermal pretreatment is capable of doubling VFA production compared to the liquid stream obtained without pretreatment. In fact, starting from 249 g of COD entering the platform, the production of VFA resulted equal to 69 gCOD with pretreatment and 31 gCOD without pretreatment. On the other hand, the transfer of organic substance into the liquid phase due to pretreatment, depleted the organic content of the residual solid stream. However, the organic substance of the residual solid fraction resulted still capable of producing significant quantities of methane.

## 4 Conclusion

Long term anaerobic operation of the reactors fed with the solid residues of FW showed always a first stable phase lasting about 2 or 3 HRTs, after which some systems showed metabolic instability, affecting methane production pathways. At HRT = 20 d, the reactor fed with thermal pretreated residue evidenced higher initial methane conversion rates (0.33 vs. 0.29 Nm^3^CH_4_ kg^-1^VS_fed_). In particular, the pretreated feed evidenced a lower disintegration constant (k_dis_ = 0.5–0.6 d^-1^) which delayed the substrate digestion, thus resulting in higher methane production over the weekends, when the reactor was not fed. However, after 50 days, the increase of hydrogenotrophic taxa in the reactor suggested the increased presence of hydrogen in the reactor thus leading to the accumulation of reduced metabolites (as VFA) and the failure of methanogenesis. The pretreated residues presented a higher soluble COD fraction, which resulted in higher methane production and higher risk of acidification when operating at lower HRTs.

On the contrary, at HRT = 40d the pretreatment did not affect the methane conversion rates, and both tests evidenced a stable phase lasting 120 days with a similar methane production of about 0.33 Nm^3^CH_4_ kg^-1^VS_fed_. After that phase, the reactor fed with untreated FW residue showed a rapid acidification together with an enrichment in acidogenic Actinobacteria members, typically related to poor methane yield in unstable digestion processes. In the reactor fed with pretreated residue, the association of hydrogenotrophic methanogens with syntrophic bacteria prevented the acidification of this system.

Modelling confirmed the eligibility of this substrate for anaerobic digestion, since the biodegradable fraction was estimated to be bounded between 75% and 100%. However, the fluctuations of the most sensitive parameters throughout the experiments suggested that the variability of the different FW stocks was the main factor influencing the process efficiency.

## Data Availability

The Dataset is available through the Sequence Read Archive (SRA) under accession PRJNA1052454. https://www.ncbi.nlm.nih.gov/bioproject/PRJNA1052454.
