# Supplementary material for: Thermally enhanced solid–liquid separation process in food waste biorefinery: modelling the anaerobic digestion of solid residues
Source: Front Bioeng Biotechnol. 2024 Feb 2;12:1343396. doi: 10.3389/fbioe.2024.1343396 (PMC10869513; doi:10.3389/fbioe.2024.1343396)
Supplement: Supplementary file 1 [file DataSheet1.docx]

Supplementary Material

# Supplementary Tables and Figures

## Supplementary Tables

Table S1: Description of the experimental tests.

|  | Substrate | Substrate COD/VS | HRT  (d) | OLR  (gVS L^-1^d^-1^) |
| --- | --- | --- | --- | --- |
| Exp1 | Raw FW | 1.17 | 20 | 1.6 |
| Exp2 | Pretreated FW | 1.35 | 20 | 1.6 |
| Exp3 | Raw FW | 1.20 | 40 | 1.6 |
| Exp4 | Pretreated FW | 1.33 | 40 | 1.6 |

Table S2: Characterization of the batch reactors before and after digestion.

|  | Raw solid residue | | Pretreated solid residue | | Blank test | |
| --- | --- | --- | --- | --- | --- | --- |
|  | Initial | Final | Initial | Final | Initial | Final |
| pH | 7.5 | 7.8 | 7.5 | 7.7 | 7.4 | 7.5 |
| TS (g/L) | 20.2±1.8 | 14.7± 1.3 | 20.2±2.1 | 15.1±1.3 | 16.3±1.2 | 14.3±1.1 |
| VS (%TS) | 72 | 61 | 72 | 64 | 64 | 62 |
| COD_tot_ (g/L) | 19.6±2.1 | 12.2±1.0 | 19.7±2.0 | 13.0±1.5 | 11.6±1.1 | 11.5±1.0 |

Table S3: Calibrated model parameters with corresponding objective functions.

|  | **NE** | | | **ME** | | | **IoA** | | | **LSE** | | |
| --- | --- | --- | --- | --- | --- | --- | --- | --- | --- | --- | --- | --- |
|  | f_xi_ | k_dis_  [d^-1^] | Value | f_xi_ | k_dis_  [d^-1^] | Value | f_xi_ | k_dis_  [d^-1^] | Value | f_xi_ | k_dis_  [d^-1^] | Value |
| **Exp1** | 0.22 | 1.5 | 0.76 | 0.20 | 1.5 | 0.75 | 0.22 | 1.5 | 0.98 | 0.22 | 1.5 | 0.05 |
| **Exp2** | 0 | 0.5 | 0.75 | 0 | 0.6 | 0.63 | 0 | 0.6 | 0.96 | 0 | 0.5 | 0.24 |
| **Exp3** | 0.15 | 1.25 | 0.66 | 0.15 | 1.5 | 0.42 | 0.15 | 1.5 | 0.91 | 0.15 | 1.25 | 0.12 |
| **Exp4** | 0.30 | 1 | 0.64 | 0.27 | 1 | 0.51 | 0.27 | 1.25 | 0.95 | 0.27 | 1 | 0.08 |

Table S4: VFAs, lactate, ethanol, and H_2_ conversion yields obtained during lab-scale acidogenic fermentation tests performed on FW liquid extracts and CH_4_ conversion yields obtained during digestion tests performed on FW solid residues.

|  | **Raw** | **TH** |
| --- | --- | --- |
|  | g/gCOD_tot_ | g/gCOD_tot_ |
| **Acetic Acid** | 0.162 | 0.118 |
| **Propionic Acid** | 0.039 | 0.013 |
| **Isobutyric Acid** | 0.015 | 0.001 |
| **Butyric Acid** | 0.123 | 0.131 |
| **Isovaleric Acid** | 0.003 | 0.001 |
| **Valeric Acid** | 0.032 | 0.076 |
| **Caproic Acid** | 0.129 | 0.074 |
| **VFAtot** | 0.505 | 0.413 |
| **Lactate** | 0.000 | 0.021 |
| **Ethanol** | 0.017 | 0.005 |
|  | **Raw** | **TH** |
|  | L/COD_fed_ | L/COD_fed_ |
| **H_2_** | 0.023 | 0.023 |
| **CH_4_** | 0.278 | 0.254 |

## Supplementary Figures


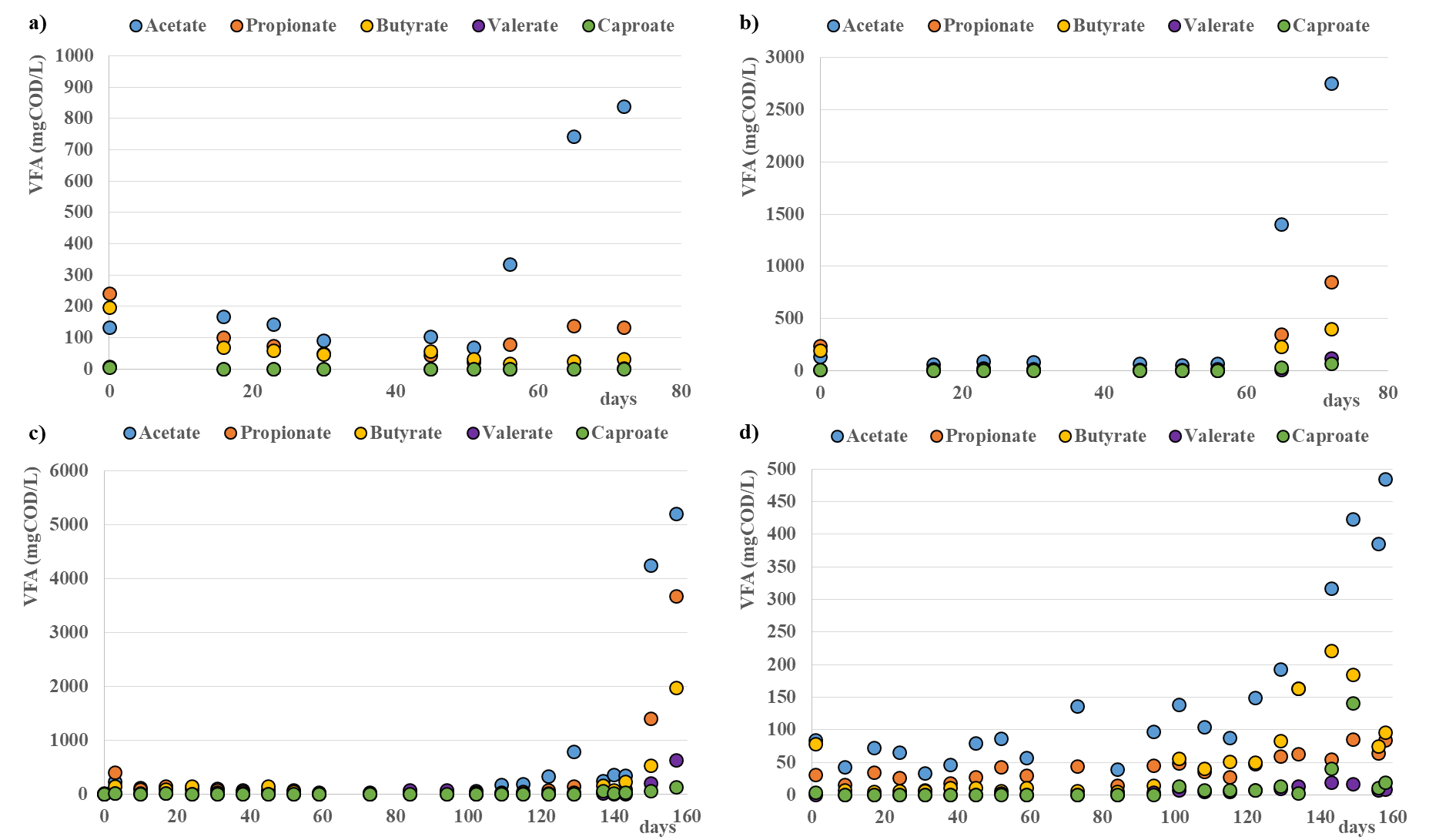


Figure S1: VFA profiles during Exp1 (a), Exp2 (b), Exp3 (c), and Exp4 (d).


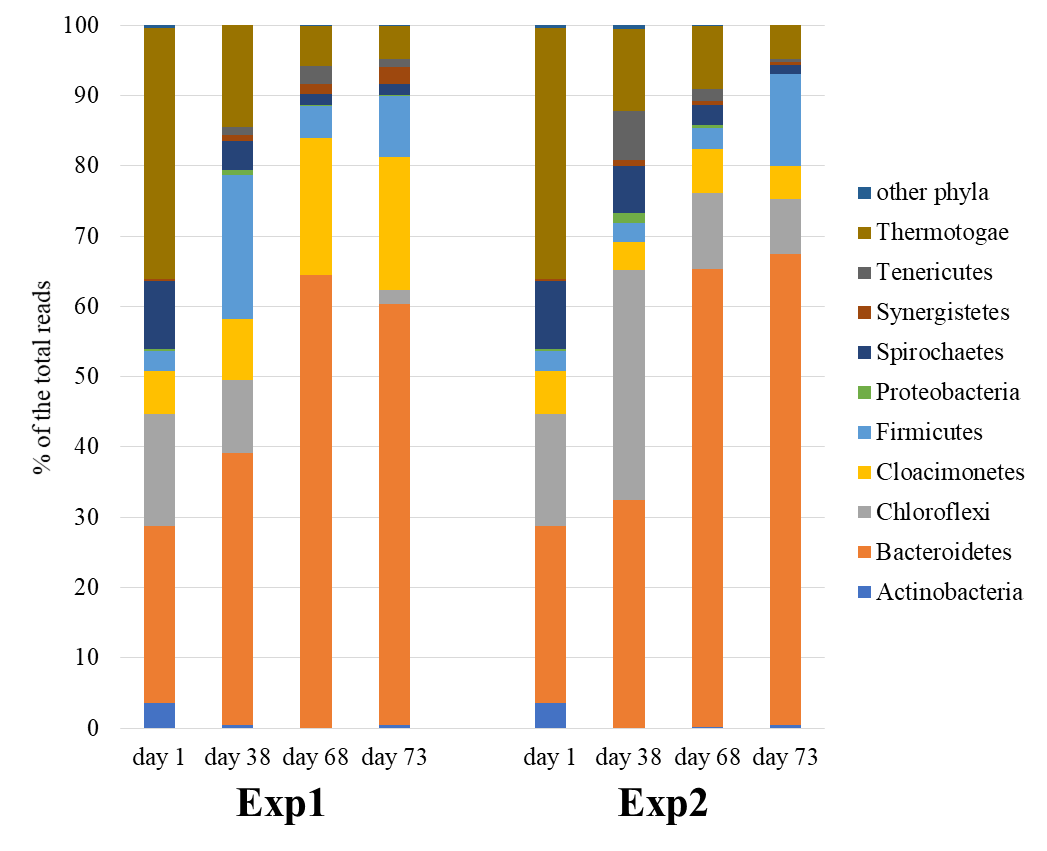


Figure S2: Bacterial composition at phylum level at different sampling times of both tests at HRT = 20d (Exp1 and Exp2)


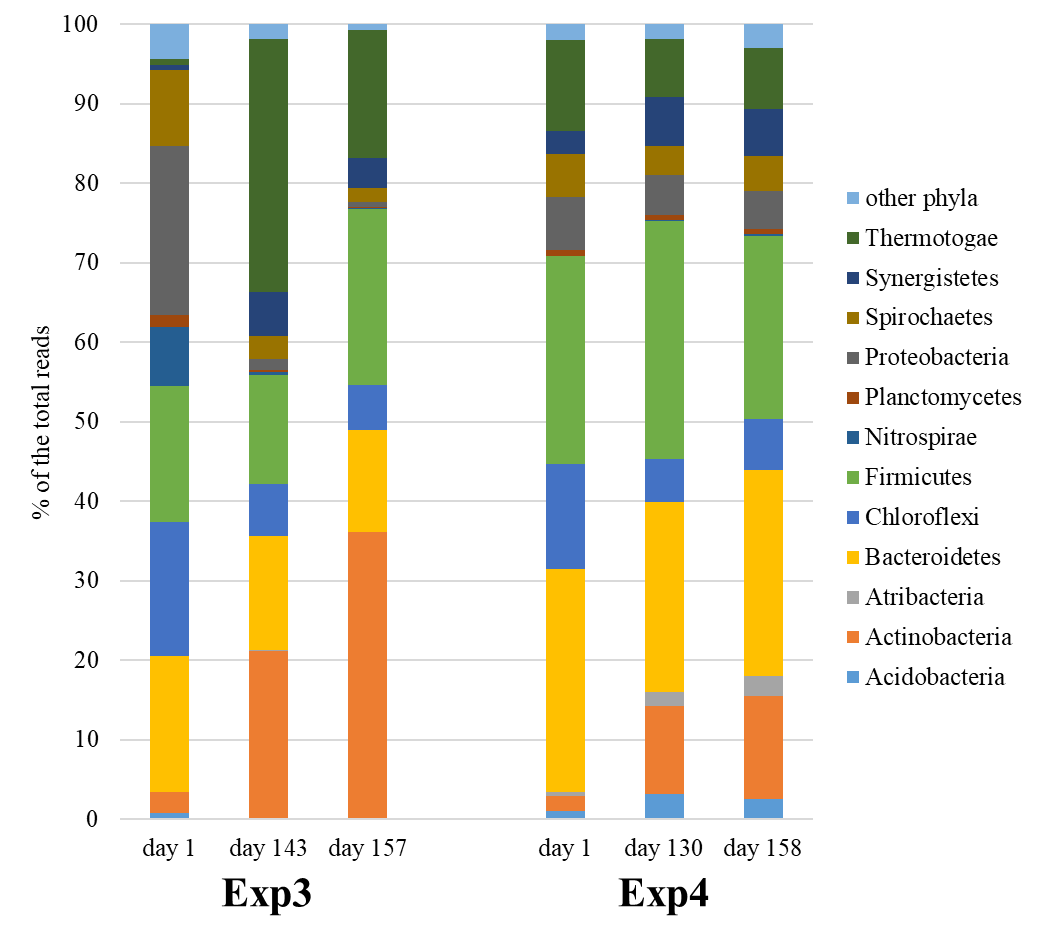


Figure S3: Bacterial composition at phylum level at different sampling times of both tests at HRT = 40d (Exp3 and Exp4).
